# Delayed lumbar plexus palsy due to giant psoas hematoma associated with vertebral compression fracture and direct oral anticoagulants: a case report

**DOI:** 10.1186/s12891-021-04267-9

**Published:** 2021-04-22

**Authors:** Chikako Ishii, Miki Komatsu, Kota Suda, Masahiko Takahata, Satoko Matsumoto Harmon, Masahiro Ota, Takamasa Watanabe, Mitsuru Asukai, Norimasa Iwasaki, Akio Minami

**Affiliations:** 1 Department of Orthopaedic Surgery, Hokkaido Spinal Cord Injury Center, Higashi-4, Minami-1, 3-1, Bibai, Hokkaido 072-0015 Japan; 2 Department of Orthopaedic Surgery, Hokkaido University Graduate School of Medicine, Kita-15 Nishi-7, Kita-ku, Sapporo, Hokkaido 060-8638 Japan

**Keywords:** Spinal fractures, Atrial fibrillation, Hematoma, Vertebral artery, Direct oral anticoagulants, Paralysis

## Abstract

**Background:**

Osteoporotic vertebral compression fractures (VCFs) are commonly observed in elderly people and can be treated by conservatively with minimal risk of complications in most cases. However, utilization of direct oral anticoagulants (DOACs) increases the risks of secondary hematoma even after insignificant trauma. The use of DOACs increased over the past decade because of their approval and recommendation for both stroke prevention in non-valvular atrial fibrillation and treatment of venous thromboembolism. It is well known that DOACs are safer anticoagulants than warfarin in terms of major and nonmajor bleeding; however, we noted an increase in the number of bleeding events associated with DOACs that required medical intervention. This report describes the first case of delayed lumbar plexus palsy due to DOAC-associated psoas hematoma after VCF to draw attention to potential risk of severe complication associated with this type of common and stable trauma.

**Case presentation:**

An 83-year-old man presented with his left inguinal pain and inability to ambulate after falling from standing position and was prescribed DOACs for chronic atrial fibrillation. Computed tomography angiography revealed a giant psoas hematoma arising from the ruptured segmental artery running around fractured L4 vertebra. Because of motor weakness of his lower limbs and expansion of psoas hematoma revealed by contrast computed tomography on day 8 of his hospital stay, angiography aimed for transcatheter arterial embolization was tried, but could not demonstrate any major active extravasation; therefore spontaneous hemostasis was expected with heparin replacement. On day 23 of his stay, hematoma turned to decrease, but dysarthria and motor weakness due to left side cerebral infarction occurred. His pain improved and bone healing was achieved about 2 months later from his admission, however the paralysis of the left lower limb and aftereffects of cerebral infarction remained after 1 year.

**Conclusion:**

In patients using DOACs with multiple risk factors, close attention must be taken in vertebral injury even if the fracture itself is a stable-type such as VCF, because segmental artery injury may cause massive psoas hematoma followed by lumbar plexus palsy and other complications.

## Background

Osteoporotic vertebral compression fractures (VCFs) are commonly observed in elderly people and are treated successfully by conservative therapy [1]. Recently, the long-term prognosis for lasting pain, disability, activities of daily living, and quality of life after VCFs has been reported [2]; however, acute, severe complications of this injury are rare and not well known.

The use of direct oral anticoagulants (DOACs) for both stroke prevention in non-valvular atrial fibrillation and treatment of venous thromboembolism increased over the past decade because of their approval and recommendation [3]. Although DOACs are also well known as safer anticoagulants as compared with warfarin in terms of major and nonmajor bleeding [4, 5], an increase in the number of bleeding events associated with DOACs that require medical intervention occurred [6, 7]. However, to our knowledge, currently, no reports exist on DOAC-related giant psoas hematoma after stable-type spinal injury.

To the best of our knowledge, this report describes the first case of lumbar plexus palsy attributed to DOAC-related secondary psoas hematoma after osteoporotic VCF. This case draws attention to the potential risk of severe complication associated with this type of common and stable trauma.

## Case presentation

An 83-year-old man presented to our hospital with left inguinal pain, slight low-back pain, and the inability to ambulate because of pain after falling from a standing position without any neurologic deficits. He presented with a medical history of chronic atrial fibrillation, carotid artery stenosis, arteriosclerosis obliterans of the lower extremities, alcoholic liver disease, and diabetes mellitus. For the treatment of atrial fibrillation, he was using the DOAC edoxaban, which is a selective, reversible, and competitive inhibitor of human factor Xa.

**Fig. 1:**
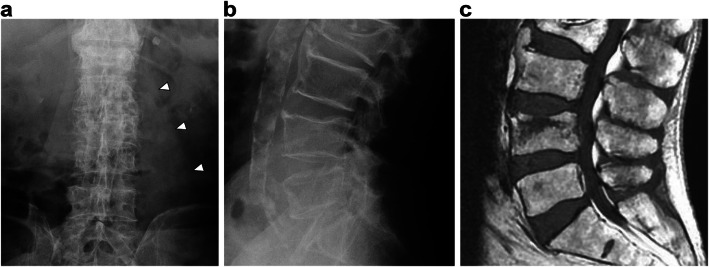
X-ray images and magnetic resonance imaging (MRI) of lumbar spine on the day of admission. **a**,**b** X-ray shows thickened left psoas major muscle (arrowhead) and no evidence of an unstable spinal fracture. **c** T1-weighted MRI shows L4 VCF estimated as a stable-type spinal injury considering that only the anterior column, and not the middle or posterior column, was injured

**Fig. 2:**
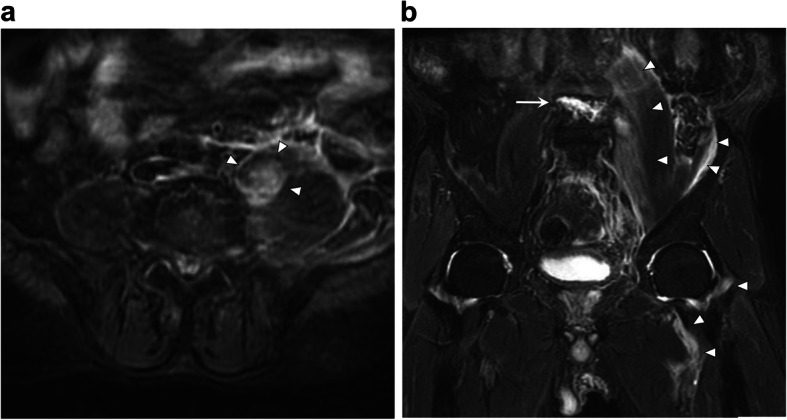
Fast STIR magnetic resonance imaging of iliopsoas hematoma. **a** Axial image shows hyperintense areas corresponding to hematoma into the psoas major muscle (arrowhead). **b** Coronal image shows the fractured L4 vertebra (arrow) and reveals that the hematoma expanded to not only the psoas muscle but also the iliac muscle and around the hip joint (arrowhead)

**Fig. 3:**
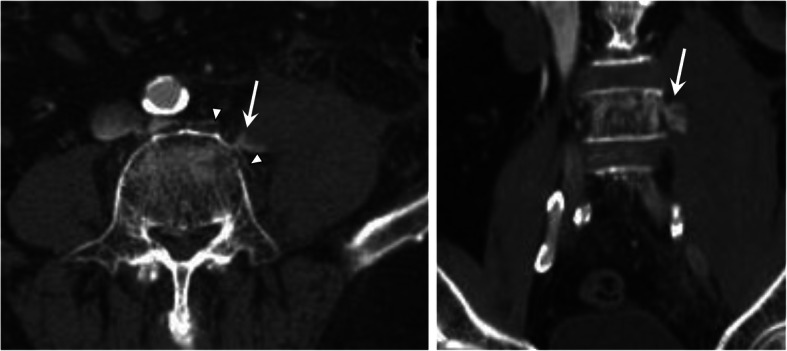
Computed tomography angiography on the day of admission. The leakage of the contrast medium (arrow) from the vertebral segmental artery (arrowhead) that runs around the fractured L4 vertebra is revealed

Radiography showed no evidence of hip fracture but revealed a thickening of left psoas major muscle, and magnetic resonance imaging (MRI) showed stable-type VCF of the fourth lumbar vertebra (Fig. 1). Moreover, fast STIR MRI revealed a 53- × 54- × 142-mm intramuscular hematoma in the psoas major muscle, which expanded to the iliac muscle and around the hip joint (Fig. 2). Computed tomography angiography showed that the hematoma arose from the ruptured segmental artery running around the fractured vertebra (Fig. 3). Blood tests showed platelets of 149,000/mm^3^ (normal, 140,000–400,000), activated partial thromboplastin time (APTT) of 30.1 s (normal, 26–40 sections), and slightly prolonged international normalized ratio of prothrombin time of 1.25 (normal, 0.90–1.10). Creatinine was 1.22 mg/dL (normal, 0.5–1.0 mg/dL), with a creatinine clearance of 44.8 mL/min.

Based on the analysis of the blood coagulation system, we estimated that extravasation from the segmental artery was encouraged not by depletion of coagulation factors due to liver disorder but by DOAC usage, which does not affect APTT. Interrupting the anticoagulant therapy was considered to pose a risk of stroke because the patient exhibited a CHADS_2_ score (defined as congestive heart failure, hypertension, age ≥ 75 years, type 2 diabetes mellitus, previous stroke [doubled]) of 3 points, and his CHA_2_DS_2_-VASc (defined as congestive heart failure, hypertension, age ≥ 75 years, type 2 diabetes mellitus, previous stroke [doubled], vascular disease, age 65–74 years, sex category) score was 5 points. Conversely, continuation of edoxaban makes decreasing hematoma expansion difficult. Therefore, we chose to treat the patient with heparin replacement with a lower therapeutic range starting the day after admission and expected spontaneous hemostasis.

**Fig. 4:**
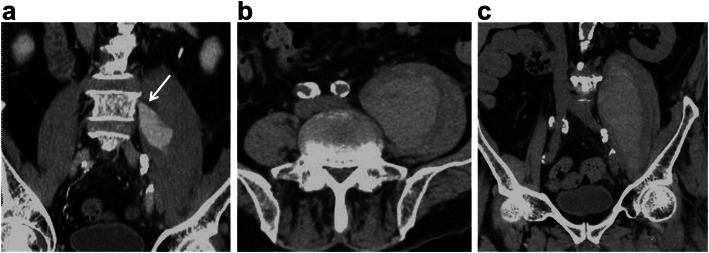
Computed tomography of developing giant psoas muscle hematoma. **a** Computed tomography angiography on day 8 after admission shows the contrast medium leakage (arrow) indicating continuation of bleeding from the vertebral segmental artery. **b**,**c** Tense swellings of the left psoas major muscle are shown in plain computed tomography on day 14 after admission

On day 8 of the patient’s hospital stay, neurologic examination revealed hypoesthesia and motor weakness of the left lower limb, evaluated as 13 of 25 points of the American Spinal Cord Injury Association (ASIA) motor score. Enhanced computed tomography showed that contrast die leaked into the hematoma from the injured segmental artery, and the psoas major muscle was tense due to a massively expanded hematoma (Fig. 4). Vascular surgery was consulted, and an angiography aimed for transcatheter arterial embolization (TAE) was performed but did not demonstrate any active extravasation; thus, TAE was abandoned. Percutaneous or surgical drainage was rejected because of the increased risk of bleeding complications and expected potential for spontaneous reduction, based on the findings of TAE. The hematoma began to decrease 1 week later.

**Fig. 5:**
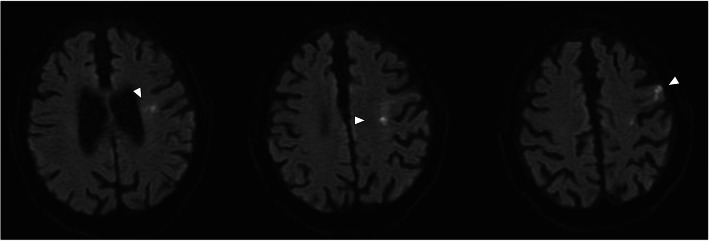
Diffusion-weighted MRI of brain. Axial diffusion-weighted MRI showing a high-signal-intensity area in the patient’s left brain, indicating acute cerebral infarction on the left cortex of the frontal lobe and radiate crown

**Fig. 6:**
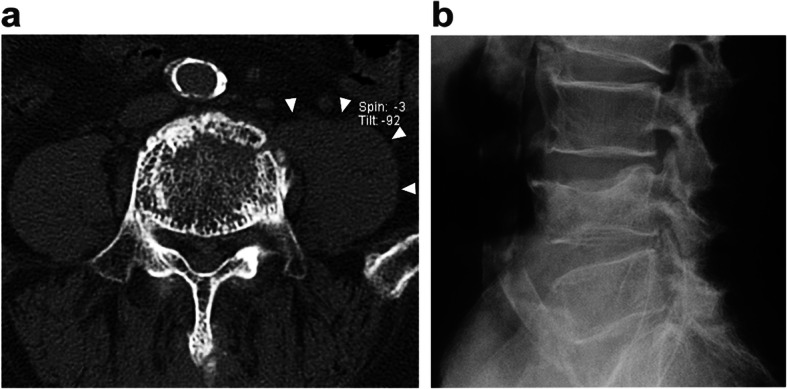
Follow-up computed tomography and X-ray image. **a** Plain computed tomography on 2 months after the patient’s hospital admission shows normal size of the psoas major muscle (arrowhead), which indicates disappearance of the hematoma. **b** X-ray at final follow-up 1 year after injury revealed bony union with minimal deformation

On day 23 of his stay, the patient experienced dysarthria and motor weakness of the right upper limb, which was evaluated as 12 points of the ASIA motor score without any sensory disturbance. A brain MRI revealed cerebral infarction on the left cortex of the frontal lobe and radiate crown (Fig. 5), and the neurosurgeon selected conservative treatment rather than thrombolytic therapy. The patient’s low-back pain and inguinal pain disappeared and bone healing was achieved; furthermore, the psoas hematoma vanished approximately 2 months after the patient’s hospital admission (Fig. 6). However, the patient experienced persistent motor weakness of the left lower limb and right upper limb as well as dysarthria, with no neurologic improvement observed at 1-year follow-up.

## Discussion and conclusions

To the best of our knowledge, this is the first report of delayed lumbar plexus palsy with DOAC-related massive psoas hematoma after osteoporotic VCF associated with the occurrence of segmental artery injury. Several reports revealed that spontaneous psoas hematoma is an infrequent complication of anticoagulant therapy [8–13]. In their retrospective multicentric study, Llitjos et al. [11] found that the occurrence of psoas hematoma was 3.01 cases/1000 admissions, and the mortality rate was 30%. Also, they revealed that 72% of the cases were related to anticoagulation therapy, including warfarin, unfractionated heparin, or low-molecular-weight heparin, except for DOACs. Ardebol et al. [14] reported the first case of spontaneous hematoma of the sartorius muscle secondary to rivaroxaban therapy; thus, DOAC-related muscular hematoma is rare. Indeed, it is much rarer for massive psoas hematoma to lead to lumber plexus palsy.

The frequency of major or clinically relevant nonmajor bleeding events related to edoxaban was reported to range from 3.8 to 19.2% [15–19]. Studies indicated that the risk factors of DOAC-related bleeding events include heavy alcohol use, uncontrolled hypertension, increasing age, heart failure, vascular disease, antiplatelet use, chronic renal failure, and diabetes mellitus [17, 20]. In our case, the patient’s age and comorbidities, including excess alcohol use, carotid artery stenosis, arteriosclerosis obliterans, and diabetes mellitus, possibly increased the risk of DOAC-related hematoma.

Lumbar segmental artery injury is usually observed in unstable injuries such as fracture dislocation [21, 22] or fracture of a patient with diffuse idiopathic skeletal hyperostosis [23]. TAE can be the treatment of choice for this arterial injury [21–26]; however, percutaneous or surgical drainage should be carefully considered because no reversal for DOAC exists, except for dabigatran, and maintaining the congealing fibrinogenolytic system is difficult [7]. In addition, emergency stabilization is effective for the prevention of secondary bleeding in cases of unstable vertebral injury [27].

Thus, in patients using DOACs with multiple risk factors, great care should be taken regarding vertebral injury, even if the fracture itself is a stable-type such as VCF, because segmental artery injury may result in massive psoas hematoma followed by lumbar plexus palsy and other complications.

## Data Availability

Not applicable.

## Abbreviations

APTT: Activated partial thromboplastin time
ASIA: American Spinal Cord Injury Association
DOAC: Direct oral anticoagulants
MRI: Magnetic resonance imaging
TAE: Transcatheter arterial embolization
VCF: Vertebral compression fractures
